# High-Resolution Thermometric Scheimpflug LiDAR for Surface Morphology and Temperature Mapping

**DOI:** 10.3390/mi16050590

**Published:** 2025-05-18

**Authors:** Xuhui Huang, Raheel Ahmed Janjua, Sailing He

**Affiliations:** 1 Centre for Optical and Electromagnetic Research, College of Optical Science and Engineering, Zhejiang University, Hangzhou 310058, China; huangxuh@zju.edu.cn (X.H.); raheel@zju.edu.cn (R.A.J.); 2 National Engineering Research Center for Optical Instruments, Zhejiang University, Hangzhou 310058, China; 3 Zhejiang Provincial Key Laboratory for Sensing Technologies, Zhejiang University, Hangzhou 310058, China; 4 Department of Electromagnetic Engineering, School of Electrical Engineering, KTH Royal Institute of Technology, SE-100 44 Stockholm, Sweden

**Keywords:** Scheimpflug LiDAR, upconversion nanomaterials, spectral detection, temperature sensing

## Abstract

Common surface temperature measurement techniques, when applied to monitoring the temperature of surfaces with complex morphology, suffer from reduced spatial resolution, which compromises the measurement accuracy of the system. To improve the spatial resolution of temperature measurement technology and maintain high temperature sensitivity, we designed a microscopic morphology thermometric LiDAR (MMTL) system based on the Scheimpflug principle, which realizes the real-time restoration of the 3D morphology and temperature of the surface of micro-structured objects. The 3D spatial resolution of the system is better than 3 μm. The theoretical resolution of the self-designed reflective spectrometer can reach 0.9 nm, which improves the sensitivity and accuracy of the upconversion hybrid nanomaterials thermometry based on the intensity ratio. In the wide temperature range of 373.15–508.15 K, the highest relative temperature sensitivity can reach 2.07%/K, the optimal temperature resolution is 0.0131 K, and the error is less than 1 K. Finally, the temperature change trend of the mold surface under different heating voltages is accurately restored. The MMTL system can provide accurate temperature distribution data and hotspot location identification for scenarios such as optimizing thermal management design and real-time risk monitoring, and it has application potential in industrial manufacturing and for electronic products.

## 1. Introduction

Temperature is a physical quantity that reflects the degree of hotness or coldness of an object, representing the average kinetic energy of its molecular motion. It is one of the most essential characteristics of a thermodynamic system. The surface of a thermodynamic system serves as a window for energy transfer with the surroundings, and its temperature limits the efficiency of this transfer. Additionally, surface temperature strongly influences material characteristics, such as crystalline structure. Sensitive and accurate surface temperature measurement systems find numerous applications in aerospace [1], energy [2], and manufacturing [3,4].

Surface temperature measurement techniques can be broadly classified into contact and non-contact methods. The most common contact temperature measurement technique is the thermocouple-based measurement, which offers rapid temperature response and a wide measurement range, making it widely used in industrial production [5]. However, due to the necessity of physical contact and relatively low precision, particularly with significant errors at low temperatures, this method is inadequate for scenarios requiring high precision and non-contact measurements [6]. Non-contact temperature measurement primarily utilizes infrared thermography, integrating infrared imaging technology with image-processing methods to achieve remote measurement, real-time imaging, and high-temperature resolution [7,8,9]. Variations in material emissivity make this method susceptible to surface reflection and environmental influences [10]. Additionally, when measuring temperatures in complex spatial structures, the relatively low spatial resolution can affect measurement accuracy, necessitating supplementary algorithmic data for improvement [11]. Distributed fiber-optic temperature sensing and microwave thermometry, despite their high measurement accuracy, are hindered by complex sensor installation and susceptibility to environmental noise, imposing numerous constraints on practical applications [12,13].

Upconversion fluorescence thermometry technology has recently witnessed numerous applications and structural advancements [14,15,16,17,18,19,20,21]. As a distinctive non-contact method for temperature measurement, this technique utilizes the fluorescent properties of upconversion materials to achieve precise temperature assessment. The underlying principle involves stimulated rare-earth elements as activators producing anti-Stokes emission, with intensity influenced by the material’s temperature, thereby enabling temperature inference. Compared to other fluorescence-based thermometry techniques, the high temperature resolution, robust stability, and broad temperature range of upconversion fluorescence [22] have garnered favor in fields demanding high precision and reliability, such as materials manufacturing [18] and biomedical applications [20,21]. Janjua et al. compared the upconversion spectra between the cubic phase (α-NaYF_4_) and the hexagonal phase (β-NaYF_4_) to enhance the upconversion luminescence intensity. They generated a heterostructure with a hexagonal phase shell on a metastable cubic core using excess Na, resulting in a 5 to 22 times enhancement in luminescence integral intensity between different core-shell structures [19]. Similarly, Ning et al. adopted a core-shell nested structure by growing a NaErF_4_:Tm^3+^ shell on NaYF_4_ cores for in vivo imaging studies, achieving a temperature resolution of 0.003 K at room temperature [17]. However, due to the significant thermal quenching effect at high temperatures in positive thermal quenching (PTQ) materials, the temperature resolution decreases significantly at high temperatures.

To mitigate the impact of thermal quenching, dual-doping of positive and negative thermal quenching (NTQ) materials can be employed. This approach utilizes the inverse changes in fluorescence peak or lattice displacement with temperature between the two materials to construct heterogeneous temperature-responsive nanoparticles. This results in sensitivity and high-temperature luminescence intensity improvements by more than one order of magnitude [15,16]. After thoroughly mixing these hybrid upconversion nanoparticles (UCNPs) with optical adhesives, various film deposition techniques, such as solution spraying, spin coating, and sputtering, were employed to create submicron-thick thermal sensing films. These films possess high sensitivity and a wide temperature range, suitable for surface temperature measurements in highly complex environments. However, their relatively low spatial resolution needs further enhancement [14].

Scheimpflug LiDAR technology (SLiDAR) is a specialized three-dimensional surface reconstruction technique, classified as a form of triangulation-based reconstruction. Integrating the Scheimpflug photographic principle with LiDAR systems ensures full-field, precise imaging of complex terrain surfaces during surface reconstruction, thereby enhancing the quality and measurement accuracy of the reconstruction. Compared to traditional Time-of-Flight (ToF) LiDAR, Scheimpflug LiDAR exhibits superior depth resolution and stability in short-range scanning applications [23]. The spectral LiDAR technology, combined with the Scheimpflug LiDAR, has been widely applied in fields such as plant detection [24,25,26,27], temperature monitoring [28,29,30], and marine analysis [31,32,33]. Zheng et al. combined a 405 nm laser diode and a color image sensor to produce a multispectral fluorescence SLiDAR system to detect plant fluorescence information, with a depth resolution of 0.7 mm [27]. Based on an SLiDAR profiler and a hyperspectral imager, Chen et al. achieved 4D hyperspectral surface reconstruction of philodendron and spider plants, with a spectral resolution better than 4 nm and a depth resolution of 0.64 mm [24]. Malmqvist et al. combined SLiDAR with dual-line atomic fluorescence temperature measurement technology and used dual-wavelength excitation light to measure the spatial temperature distribution of indium-doped flames in burners [30].

Here, we designed and built a microscopic morphology thermometric LiDAR (MMTL) system. To the best of our knowledge, this represents the first integration of spectral LiDAR technology based on the Scheimpflug principle with UCNPs temperature measurement techniques, enabling simultaneous reconstruction of surface morphology and the temperature of objects. The system consists of a morphology optical path based on the Scheimpflug principle and a reflection imaging spectrometer. Two sets of cylindrical lenses were used to shape and widen the 976 nm point laser, and a dichroic mirror was added to make the morphology line laser and the slit of the imaging spectrometer conjugate, ensuring that the surface morphology information and spectral information of the object could be collected simultaneously. Subsequently, the spatial coordinate calibration equation was used to map and match the imaging information of the two optical paths to achieve the recovery of the fluorescence morphology model of each point on the surface of the object.

In addition, we mixed NaYF_4_ Core-Shell Nanocrystals with PTQ effect and Tm-doped Yb_2_W_3_O_12_ nanoparticles with NTQ effect together with an optical adhesive and sprayed them onto the surface of objects to form submicron temperature-sensing films. The system achieved a maximum depth resolution of 2.1 μm and the theoretical spectral resolution was 0.9 nm. Within the temperature range of 373.15 K–508.15 K, the system exhibited a relative temperature sensitivity of up to 2.07%/K and the temperature uncertainty was less than 1 K. Subsequently, we reconstructed the surface morphology and temperature of the standard block gauge and metal mold to verify the recovery performance of the system, revealing its broad application prospects in industrial manufacturing and other fields.

## 2. Principle

### 2.1. Scheimpflug Principle

The Scheimpflug principle states that when the object plane, the equivalent lens plane, and the image plane are not parallel to each other and intersect on the same line, the system can theoretically achieve an infinite depth of field [34]. The MMTL system uses this principle to achieve the inversion calculation of depth and height information with respect to the pixel coordinates under a large depth-of-field.

**Figure 1:**
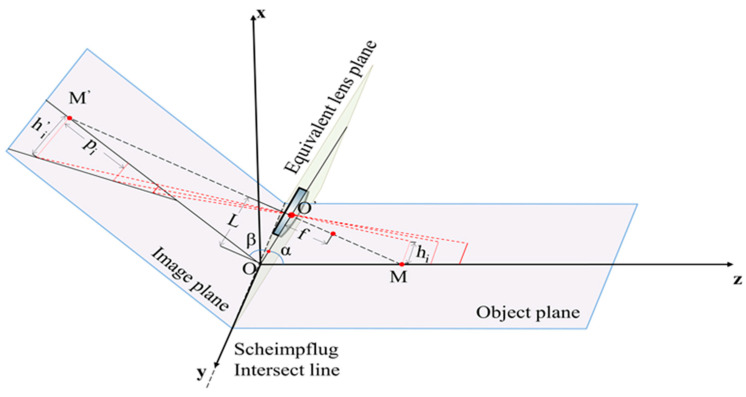
The Scheimpflug principle in 3D space. O′ is the center point of the equivalent lens, O is the origin of the coordinates obtained by projection, f is the focal length, L is the baseline length, α is the angle between the lens plane and the object plane, and β is the angle with the image plane; the optical axis intersects the object plane at M and intersects the image plane at M′. h_i_, h_i′_ are the heights of the object and its corresponding image during calibration, respectively. The lines with different transparency represent objects at different distances.

The 3D spatial model of the Scheimpflug principle is shown in Figure 1. The coordinate system is established with the projection point of the equivalent lens plane center on the intersection line as the origin, the *z*-axis perpendicular to the Scheimpflug intersection line and along the object plane direction, and the *y*-axis parallel to the Scheimpflug intersection line. The depth from the origin to the center of the equivalent lens is called the baseline length L, α is the angle between the lens plane and the object plane, and β is the angle with the image plane. The optical axis of the equivalent lens intersects the object plane at point M and intersects the image plane at point M′. The relationship between the depth *z* from any object point on the *z*-axis to the origin O and the relative distance *p_I_* on the image plane can be obtained with the following equation [32,35]:

(1) $$z=\frac{\mathrm{Lcsc}\alpha (p_{I}\sin\beta +\mathrm{Ltan}\beta )}{p_{I}(\cos\beta +\sin\beta \cot\alpha )+\mathrm{Ltan}\beta \cot\alpha }=\frac{Ap_{I}+B}{Cp_{I}+D}$$

Here, L is the baseline length and α, β are the angle between the three planes. A, B, C, D are constant coefficients. *p_I_* is the distance from the image point to the reference point M′ and is the only variable. Assume that *N_i_* and N_M′_ are the pixel index values of the image point and the reference point M′ on the image sensor, respectively, and ω is the camera pixel size. *p_I_* can be expressed as:

(2) $$p_{I}=\left(N_{i}-N_{M^{\prime }}\right)\omega$$

Now we combine Equations (1) and (2), and separate the variable in the molecule, simplifying them into a constant term. After extracting the constant term, Equation (1) can be simplified to a hyperbolic function [24]:

(3) $$z=\frac{A}{C}+\frac{B-\frac{AD}{C}}{C\omega \left(N_{i}-N_{M^{\prime }}\right)+D}=C_{1}+\frac{C_{4}}{C_{2}N_{i}+C_{3}}$$

where *N_i_* is the pixel index values of the image point. C_1_, C_2_, C_3_, and C_4_ are constant coefficients, which can be obtained by fitting the depth calibration data.

The height information of any object point on the object plane can be calculated by inverting the longitudinal axis magnification corresponding to different pixel points [36]. As shown in Figure 1, the calibration of the longitudinal axis magnification requires taking multiple object lines parallel to the Scheimpflug intersection line on the object plane, which has the same height information, *h_i_*, and different depth information. *h_i_*′ is the height information of the image line corresponding to each object line. Linear fitting is performed on the beginning and end of each image line, respectively, and the distance between the two fitting lines is the image height corresponding to each pixel. Then, using the lens imaging principle, we can obtain the relationship between the short-axis magnification, *β_i_*, and the difference between the first and last short-axis pixel index values Δ*N_i_*:

(4) $$\beta_{i}=\frac{{h_{i}}^{\prime }}{h_{i}}=\frac{\omega \Delta N_{i}}{h_{i}}$$

After the calibration of the longitudinal magnification, we can obtain the longitudinal magnification function *β* (*N_i_*). Given the depth information, *z_i_*, of any point in the object plane and the height information, *h_i_*′, of the corresponding image point, the corresponding index value *N_i_* can be obtained according to Equation (3). The object height information of the point can be obtained by using Equation (4).

### 2.2. Upconversion Primary Thermometry

The narrow excitation peak, large absorption-to-emission spectrum energy difference, and long luminescence lifetime have made lanthanide-doped upconversion nanoparticles (UCNPs) a common material in the field of contactless temperature sensing [17,22]. UCNPs are a host-guest system material that achieve anti-Stokes scattering under near-infrared (NIR) light excitation by doping trivalent lanthanide ions (Ln^3+^) in the lattice of dielectric host materials [37].

The ions in the dielectric host material have a large absorption cross section in the NIR band, which allows the low-energy photons absorbed from the NIR excitation light to transition non-radiatively to the step-distributed energy levels of nearby Ln^3+^, thereby generating wide-band anti-Stokes emission. Since the thermally coupled energy level difference between the two nanomaterials lies within the range of 200~2000 cm^−1^, under thermal excitation, the electrons of the two levels follow the Boltzmann distribution. The luminescence intensity of UCNPs (*I_T_*) can be fitted by the absolute temperature *T* [14]:

(5) $$I_{T}=I_{0}[A_{0}\exp(B_{0}/T)+C_{0}]$$

Here, I_0_ is the luminescence intensity of UCNPs at 0 K; A_0_, B_0_, and C_0_ are fitting parameters related to the characteristics of the doped lanthanide ions; and *T* is the absolute temperature.

As the temperature increases, the intensity decreases exponentially, and UCNPs exhibit a positive thermal quenching effect (PTQ). The partial energy of UCNPs will be dissipated in the form of heat energy, such as multi-phonon relaxation, and the fluorescence lifetime will be sharply shortened at high temperature, posing a considerable challenge to detection accuracy and error control. To improve the relative thermal sensitivity of UCNPs under high-temperature conditions, a heterostructure can be formed by simultaneously introducing materials with a negative thermal quenching effect (NTQ). Since PTQ materials usually have thicker shells, the energy transfer and cross-relaxation between the ions doped in the PTQ core and the ions doped in the NTQ material can be neglected. Therefore, the fluorescence intensity ratio of the mixed material is simply the ratio of the individual emission intensities. In Equation (6), the fitting relationship between the fluorescence emission peak intensity ratio *FIR* and the absolute temperature *T* can be expressed as [14,15]

(6) $$FIR=\frac{I_{T,P}}{I_{T,N}}=A\exp(B/T)+C$$

where *I_T_*_,_*_P_*, *I_T_*_,_*_N_* are the emission peak values of the PTQ material and NTQ material at the unique variable temperature *T*, respectively, and A, B, C are fitting parameters. However, this fitting model is not applicable for single-core structures or when the shell layer is very thin.

## 3. System and Calibration

### 3.1. System Setup

**Figure 2:**
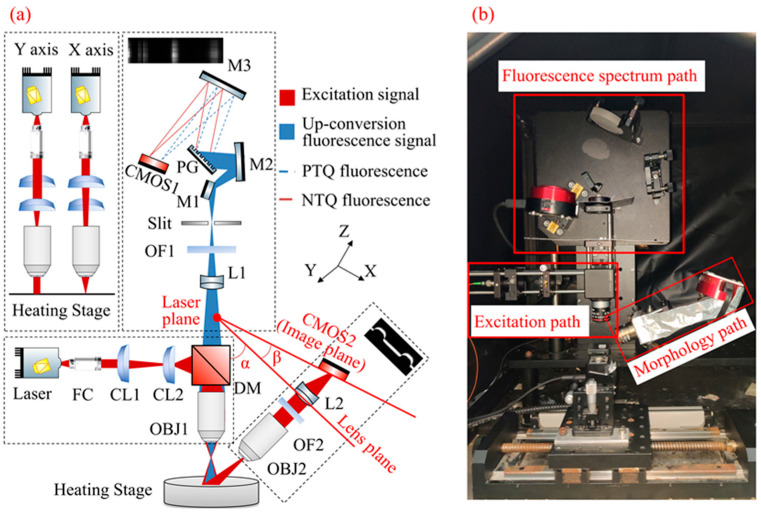
Structural diagram of the microscopic morphology thermometry LiDAR (MMTL) system. (**a**) Optical path of MMTL system. M, mirror; L, lens; FC, fiber collimators; CL, cylindrical lens; DM, dichroic mirrors; OBJ, objective; OF, optical filter. (**b**) Photography of the MMTL system.

The MMTL system structure is shown in Figure 2; it consists of three parts: the line light source excitation path, the morphology information path, and the fluorescence spectrum information path. The system light source uses a 976 nm commercial infrared laser (DS3-51412-0711, BWT Beijing Ltd., Beijing, China) and is coupled with the linear laser excitation path through a multimode optical fiber. Its maximum output power is 50 W and its wavelength shift is < 0.03 nm. The laser power used in this study was fixed at 2 W. The line laser excitation path consists of fiber collimator FC (F280APC980, Thorlabs, Newton, NJ, USA); f = −150 mm cylindrical lens CL1 (LK1743L1-B, Thorlabs, USA) and f = 50 mm CL2 (LJ1695RM-B, Thorlabs, USA). A 900 nm short-pass dichroic mirror (DMSP900, Thorlabs, USA) and a 4X microscope objective OBJ1 (CFI E Plan Achromat 4X, Nikon, Shinagawa-ku, Japan) were used. The spot diameter after FC collimation was about 5 mm, and the X, Y directions of the laser were, respectively, shaped by CL1 and CL2, as shown in the upper left black box of Figure 2a. Finally, OBJ1 focused the broadened line laser onto the object surface on the heating stage, and the height of the heating stage could be adjusted. We could adjust the position of CL1 to achieve precise focusing of the laser within ±4 mm distance, and its width was ~20 μm.

The morphology information path includes the 4X microscope objective lens OBJ2 (CFI Achro 4X, Nikon, Japan), the doublet lens f = 100 mm L2 (145058, Grand Unified Optics, Beijing, China), the 980 nm narrow-band optical filter OF2 (FBH980-10, Thorlabs, USA), and the area array sensor CMOS2 (ASI174MM, 1936 × 1216 pixels, 5.86 μm × 5.86 μm, ZWO, Suzhou, China). The reflector laser on the object surface was captured by OBJ2 and focused through L2 on the image plane CMOS2 with an inclination of 44.53°. The laser plane (object plane), lens plane, and image plane satisfied the Scheimplug principle. The depth and height information of the area irradiated by the line laser on the object surface was captured by CMOS2. The long-axis corresponded to the depth information of each point (*z*-axis), and the short-axis corresponded to the height information (*y*-axis). Finally, OF2 was used to filter out noise signals and improve the signal-to-noise ratio (SNR).

This fluorescence spectrum information path includes a f = 80 mm doublet lens L1 (145057, Grand Unified Optics, Beijing, China), an 850 nm low-pass filter OF1 (FESH0850, Thorlabs, USA), a plane reflector M1, concave reflectors M2 (CM508-100-E02, Thorlabs, USA), M3 (CM508-100-E02, Thorlabs, USA), a plane reflection grating PG (#37-118, Edmund, NJ, USA), and an area array sensor CMOS2 (ASI1600MM, 4656 × 3520 pixels, 3.8 μm × 3.8 μm, ZWO, Suzhou, China). The fluorescence signal was collected by OBJ1 and focused on the slit (30 μm wide) through L1 with the filtering of OF1. Then M2 collimated the fluorescence signal and sent it to the PG. The PG separates the fluorescence peaks of the two thermal quenching materials and focuses them on CMOS1 through M3. The long-axis of the sensor corresponded to the spectral information of each point (λ-axis), and the short axis corresponded to the height information (*y*-axis).

The images collected by each path were attached to their box. We obtained three-dimensional information (y, z, λ) about the area irradiated by the line laser on the object surface. The width information (*x*-axis) can be obtained by push-broom scanning along the vertical linear region with a uniaxial moving controller (KSA150-11-X, Zolix, Beijing, China) to recover the morphology and fluorescence information of each point on the surface.

### 3.2. Sample Preparation

Synthesis Method for Core/Shell Nanocrystals: For the synthesis of the NaYF_4_: 20Yb/2Er@NaYF_4_ core-shell nanocrystals, 1 mM of Y(Ac)_3_ was added to a mixture of 10 mL oleic acid and 10 mL 1-octadecene. The solution was heated to 150 °C for 30 min, during which a clear solution formed. A methanol solution of NH_4_F and NaOH was then added to the mixture at room temperature. The NaYF_4_: 20Yb/2Er nanocrystals, prepared according to the reported procedure14, were used as cores. A total of 1 mM of cores particles were dispersed in hexane and added to the mother solution and stirred to ensure a homogeneous mixture. The resulting solution was heated to 70 °C until all the hexane was removed, and then the temperature was raised to 290 °C under a nitrogen atmosphere and maintained at this temperature for 120 min, allowing the shell to grow uniformly around the core. After the reaction, the mixture was cooled to room temperature. The Core-Shell Nanocrystals were precipitated using ethanol, washed thoroughly to remove unreacted precursors and by-products, and stored for further characterization and use.

Synthesis of Tm^3+^ Doped Yb_2_W_3_O_12_ Bulk Products: For the synthesis of Yb_2_W_3_O_12_, Yb_2_O_3_ (0.985 mmol), Tm_2_O_3_ (0.015 mmol), and H_2_WO_4_ (3 mmol) were thoroughly mixed in a ball mill. The resulting powder was placed in a tungsten crucible and heated to 1000 °C at a rate of 10 °C/min in a lifting furnace, maintaining this temperature for 6 h in an atmospheric environment. The sample was then allowed to cool naturally to room temperature.

Preparation of colloidal suspension: Colloidal suspension was prepared with the following stoichiometry: NaYF_4_:20Yb/2Er@NaYF_4_:Yb_2_W_3_O_12_:HPC = 1:4:5. Initially, NaYF_4_: 20Yb/2Er@NaYF_4_ and Yb_2_W_3_O_12_ (1.5% Tm) nanocrystals were mixed and ground for 30 min to achieve a consistent powder. Hydroxypropyl cellulose (HPC) was added, and the mixture was blended for another 30 min. The resultant mixture was then dissolved in an appropriate volume of ethanol to create a homogeneous solution. The solution underwent ultrasonic treatment for 24 h to ensure stability and prevent agglomeration. After ultrasonication, the suspension was allowed to rest, and the supernatant was carefully collected for further characterization and use. Detailed information on sample characterization and film preparation can be found in the Supplementary Material.

### 3.3. Distance and Height Calibration of Morphology Information Path

#### 3.3.1. Morphology Depth Calibration (*z*-Axis)

The imaging module of the morphology information path of this system is composed of a microscope objective lens OBJ2 and a doublet lens L2, whose focal lengths are 50 mm and 100 mm, respectively, and the spacing is 22 mm. Using the multi-light group imaging law, the equivalent lens focal length f is 38.6 mm and the baseline length L is 140 mm. In addition, the system’s intrinsic parameters are as follows: α (25°), β (44.5°), N_M′_ (205), and ω (5.86 μm). According to Equation (1), the theoretical relationship curve between the long-axis pixel index value in the CMOS2 sensor and the depth can be obtained, which is used as a reference for the subsequent fitting relationship curve.

**Figure 3:**
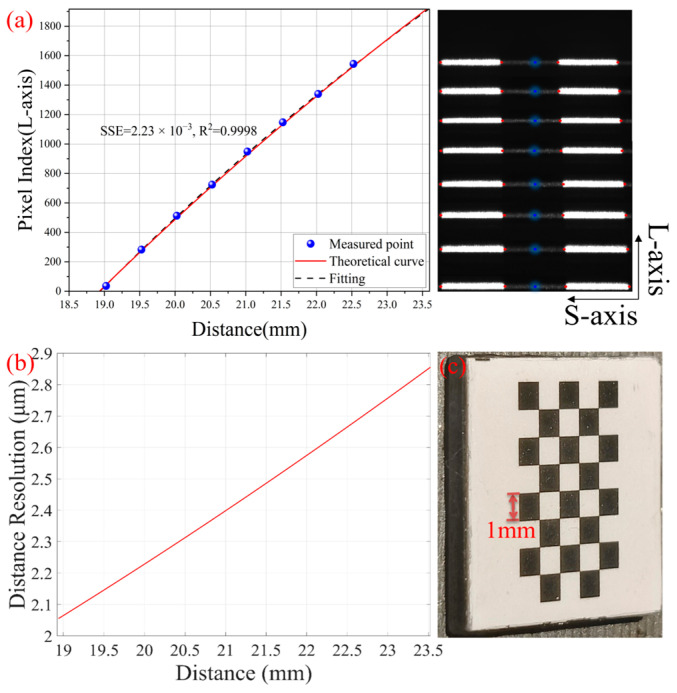
Morphology path depth calibration (*z*-axis). (**a**) Pixel-depth relationship and spliced calibration image. (**b**) Depth resolution of the morphology path. (**c**) Small standard chessboard.

We used a small standard chessboard for the morphology path fitting calibration, as shown in Figure 3c. The size of the chessboard was 10 mm × 10 mm, consisting of 8 × 5 squares, and each square had a side length of 1 mm. The laser exposure time in the *z*-axis calibration was 50 ms. Since the light sheet formed by the line laser was perpendicular to the heating stage, we placed the chessboard on the heating stage and adjusted the height of the heating stage to obtain different depth information (the adjustment step was 0.5 mm). We spliced the calibration images of eight different depths (19, 19.5, 20, 20.5, 21, 21.5, 22 and 22.5 mm) for subsequent fitting, as shown in Figure 3a. As the depth changes, the laser stripes move horizontally along the long-axis direction of the sensor. Each stripe contains the laser line information of three squares (due to different reflectivity, white grids appear as bright lines and black grids appear as dark lines). Four bright and dark intersection points (red dots) were selected and their long-axis pixel coordinates were averaged (blue dots) to obtain the long-axis pixel index value of the corresponding depth (36, 283, 513, 724, 949, 1147, 1340, and 1544). The two sets of data were fitted using Equation (3), and the fitting results are shown in Figure 3a. The fitting curve is consistent with the theoretical curve. The sum of squared errors (SSE) was used to assess model fitting performance, with smaller values indicating better fitting.

Using the depth information *z* to derive *N_i_*, the corresponding relationship between the system depth resolution and the depth can be obtained, as shown in Figure 3b. In the range of 18.9 mm to 23.5 mm, the system depth resolution increases as the depth decreases, up to 2.1 μm.

#### 3.3.2. Morphology Height Calibration (*y*-Axis)

**Figure 4:**
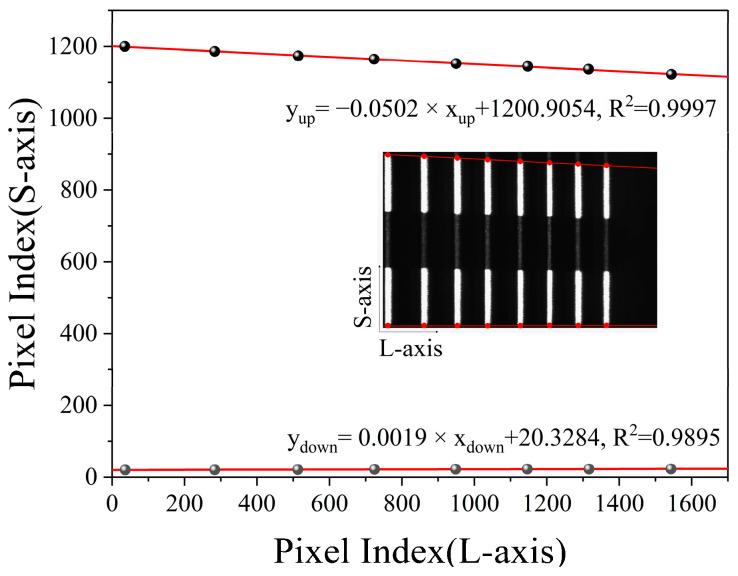
Morphology path height calibration (*y*-axis) with the fitting lines of edge points and spliced calibration image.

After obtaining the relationship between the pixel index value and the depth, we can refer to Section 2.1 to calibrate the height magnification corresponding to each pixel index value. As shown in Figure 4, we also spliced the eight images collected in the previous section, then marked the edge intersection points of each stripe (red dots) and fit the marked points on the upper and lower edges, respectively. The fitting equations are y_up_ = −0.0502 × x_up_ + 1200.9054 and y_down_ = −0.0019 × x_down_ + 20.3284, both of which have good linearity. As shown in Figure 1, the intersection of the two fitting lines and the reference point M′ have the same short-axis pixel index value on the sensor (63), which represents the index value of the height baseline.

**Figure 5:**
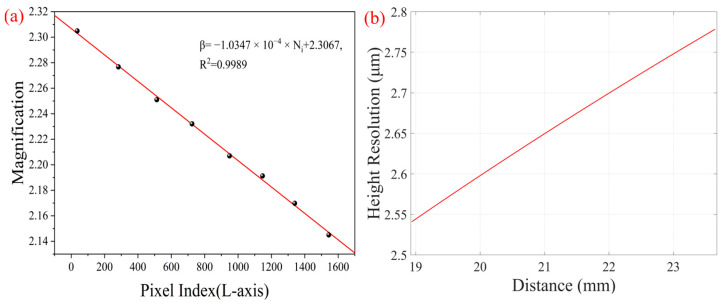
Height calibration results of the morphology path and its resolution capability. (**a**) Fitting result of magnification; the black points are the magnification of measuring depth; the red line is fitting line. (**b**) High resolution result.

Next, we calculated the difference between the short-axis pixel index value of each stripe edge point Δ*N_i_* (−1180, −1166, −1152, −1143, −1130, −1122, −1115, and −1099) and the long-axis pixel index value (36, 283, 513, 724, 949, 1147, 1340, and 1544). According to the system parameters ω (5.86 μm) and h_i0_ (3 mm), the magnification *β* (*N_i_*) corresponding to each long-axis pixel can be fitted by using Equation (4). The fitting equation is *β* (*N_i_*) = −1.0347 × 10^−4^ × *N_i_* + 2.3067, as shown in Figure 5a. If we assume that the pixel coordinate of an object is (*N_i_*, *M_i_*), whose height information on the object plane is *h_i_*, then the true height *h_i_* can be obtained by using the following equation:

(7) $$h_{i}=\frac{\left(M_{i}-63\right)\omega }{\beta (N_{i})}$$

where ω is the pixel size of CMOS2, and *β* (*N_i_*) is the magnification corresponding to each long-axis pixel. We substituted the pixel value of the upper edge at each depth into the Equation (7) and obtained the distance from the upper edge of the chessboard to the object axis (*z*-axis), which is 0.1056 mm. In addition, the relationship between the system height resolution and long-axis pixels is shown in Figure 5b. In the range of 18.9 mm to 23.5 mm, the resolution of the system becomes higher as the depth decreases, up to 2.53 μm.

### 3.4. Height and Temperature Calibration of Fluorescence Spectrum Information Path

#### 3.4.1. Height Calibration (*y*-Axis)

For the chessboards at different depths described in the previous section, we simultaneously collected the corresponding spectral information for the height calibration. The long- and short-axes on the CMOS1 sensor of the imaging spectrometer system correspond to the wavelength information and height information of the object line, respectively. Therefore, we can sum the spectral information along the long-axis direction to obtain the intensity values at different heights on the target line at a certain depth.

**Figure 6:**
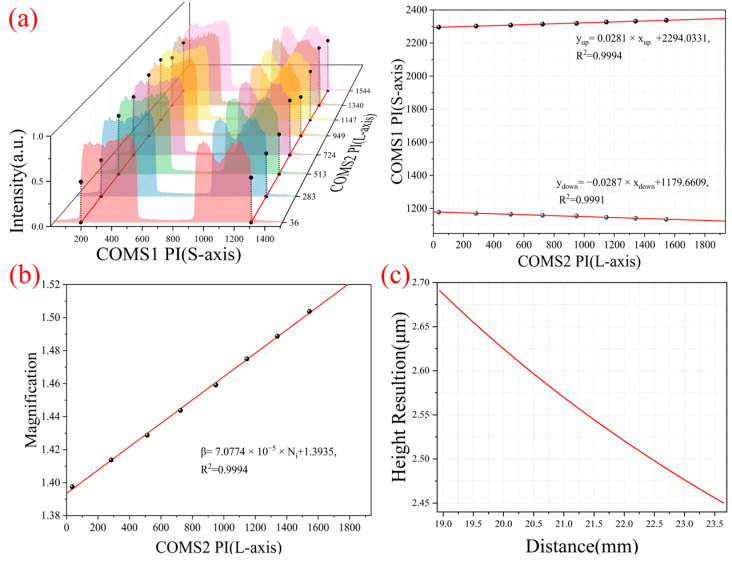
Spectral path height calibration (*y*-axis); (**a**) edge intensity mutation point selection and fitting lines. (**b**) Fitting result of spectral path magnification; the black points are the magnification of measuring depth; the red line is a fitting line. (**c**) High resolution of the spectral path.

We adopted a height calibration method like the morphology path. Since the reflection intensity of the white squares on the chessboard was significantly higher than that of the black squares, the intensity mutation points on both sides were selected as the edge points required for calibration. In addition, the two channels of height information were collected simultaneously to ensure that they corresponded to the same depth information and CMOS2 long-axis pixel index value (PI). We spliced the set of fluorescence information and linearly fit the edge points, as shown in Figure 6a. The fitting equations are *y_up_* = 0.0281 × *x_up_* + 2294.0331 and *y_down_* = −0.0287 × *x_down_* + 1179.6609. Using the intersection of the two fitting lines, we could obtain the short-axis index value (1738) of the height reference line in the spectral path.

Similarly, the image height *h_i_* and magnification *β_i_* of CMOS1 at different depths were calculated using Equation (4) and then fitted with the long-axis PI of CMOS2 to obtain the magnification *β_S_* of CMOS1 corresponding to each long-axis PI of CMOS2. The fitting equation is *β_S_* = 7.0774 × 10^−5^ × *N_i_* + 1.3935, as shown in Figure 6b. For an object whose height information on the object surface is *h_i_*, the short-axis pixel imaged on CMOS1 is *M_Si_*, and the pixel coordinate imaged on CMOS2 is (*N_i_*, *M_i_*), and then the height *h_Si_* from the spectral path axis can be obtained by the following equation:

(8) $$h_{Si}=\frac{\left(M_{Si}-1738\right)\omega_{S}}{\beta_{S}(N_{i})}$$

Here, ω_S_ is the pixel size of CMOS1 (3.5 μm), and *β_S_* (*N_i_*) is the magnification corresponding to each long-axis pixel. We also substituted the upper edge pixel value at each depth into Equation (8), and we calculated that the distance from the upper edge of the chessboard to the optical axis of the spectral path is 1.5044 mm, and the optical axis distance between the two paths is 1.3988 mm. Furthermore, the relationship between the height resolution of the spectral path and the long-axis pixel of CMOS2 is shown in Figure 6c. The height resolution of the spectral path increases with depth, with a maximum resolution better than 2.7 μm.

#### 3.4.2. Spectral and Temperature Calibration (λ-Axis)

To improve the system acquisition efficiency and eliminate the influence of chromatic aberration, we improved the M-type Czerny-Turner imaging spectrometer and designed a concentric reflective structure using OpticStudio (Zemax LLC, Kirkland, WA, USA). Next, we performed spectral calibration. Since the grating’s spectral direction is along the long axis of CMOS1, we needed to calibrate the relationship between the long-axis pixel index value and the wavelength.

**Figure 7:**
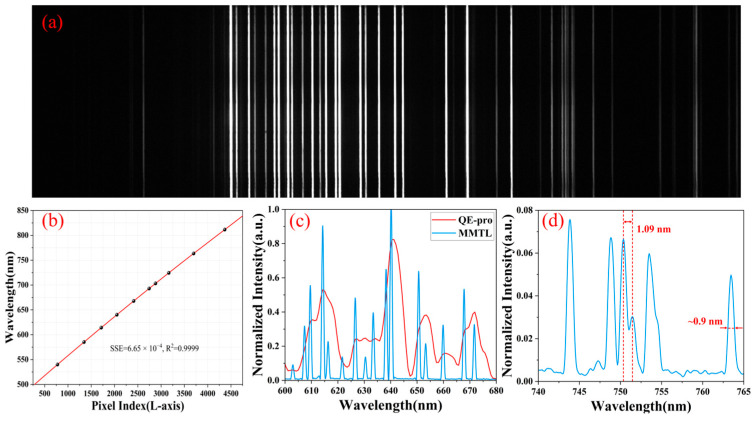
Spectral calibration (λ-axis). (**a**) Neon calibration light source’s spectrum collected by MMTL system. (**b**) Calibration results between the wavelength and long-axis pixel index. (**c**) Comparison of the spectrum between the MMTL System and the QE-pro. (**d**) Measured spectrum of the MMTL system; theoretical resolution is up to 0.9 nm.

A neon calibration light source (NE-1, Wyoptics, Shanghai, China) was used for spectral calibration. The imaging result of the spectrometer is shown in Figure 7a, and the characteristic peaks of the light source are sharply separated. We chose 10 spectral lines (540.06 nm, 585.25 nm, 614.31 nm, 640.23 nm, 667.83 nm, 692.95 nm, 703.24 nm, 724.51 nm, 763.51 nm, 811.53 nm) and their corresponding long-axis pixel index value *N_i_* (776, 1345, 1716, 2051, 2411, 2743, 2880, 3166, 3699, 4367) to calibrate; the calibration results are shown in Figure 7b. The relationship between the two can be obtained by fitting a fifth-order polynomial equation [38,39].

Using this calibration result, we restored the spectrum of the neon calibration light source and compared it with the commercial reflection spectrometer (QE-Pro, Oceansight Inc., Orlando, FA, USA), as shown in Figure 7c. With the same grating frequency and the slit width, the aliasing spectral lines measured by the commercial spectrometer were sharply distinguished by the MMTL system (with four groups of characteristic peaks identified within the red envelope from 605 nm to 620 nm), significantly enhancing the spectral resolution capability. As shown in Figure 7d, the MMTL system can clearly distinguish the two characteristic peaks at 750.35 nm and 751.44 nm, and the FWHM of the characteristic peak at 763.44 nm is approximately 0.9 nm. The theoretical spectral resolution of the system can reach 0.9 nm.

After completing the spectrum calibration, we placed the standard block gauge on a heating stage (MPH30, e-Design Co., Ltd., Guangzhou, China) and we used an air brush to spray the colloidal suspension prepared in Section 3.2 on its surface. The temperature of the heating stage was set to 100 °C for rapid film solidification. The block gauge thickness was 1 mm, and its high flatness improved the uniformity of the film layer, and its good thermal conductivity reduced the impact of its temperature gradient on the calibration results. After that, the film layer was left at room temperature for 1 h and then heated at 150 °C for 20 min to improve its adhesion. The heating area of the heating stage was 30 mm × 30 mm, and the temperature stability within the heating range of 100–350 °C was 3%. The temperature calibration range was selected as 373.15 K–508.15 K (100–235 °C), the step size was 15 K, and it was stabilized for 15 min after each temperature change. At each calibration temperature, the spectrum information and the background information were repeatedly collected 10 times. After deducting the background information, the spectrum information was averaged to obtain the fluorescence information at that temperature. The intensity information of each row in the image corresponds to the fluorescence spectrum at different heights on the surface of the film. The laser exposure time in the temperature calibration was 150 ms. The Supplementary Material states that the heating effect of the laser does not affect the temperature results.

**Figure 8:**
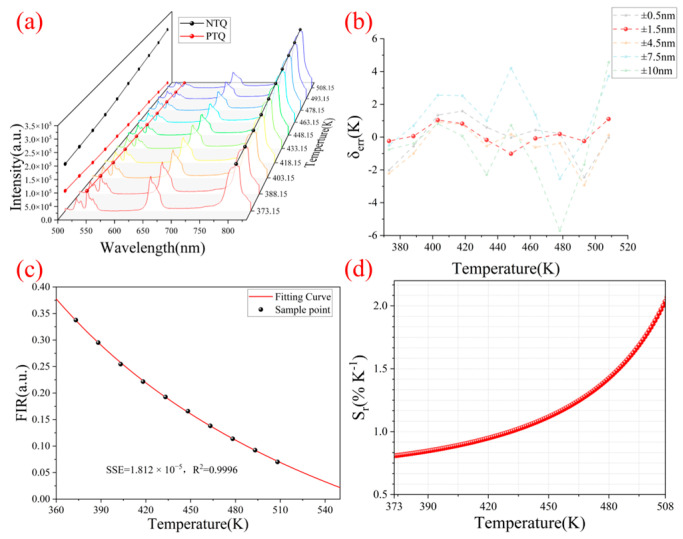
Temperature calibration. (**a**) Temperature-dependent fluorescence spectrum of the central pixel. (**b**) Temperature-related errors under different filter bands. (**c**) Relationship between fluorescence intensity ratio and temperature in the ±1.5 nm filter band. (**d**) Relative temperature sensitivity changes with temperature.

The trend of the fluorescence spectrum in the center row of the image (pixel index value is 1760) with temperature is shown in Figure 8a. The opposite trends of the characteristic peak at 539 nm of the positively quenched material and the characteristic peak at 806 nm of the negatively quenched material confirms the theory in Section 2.2. Since temperature changes cause spectral peak changes and spectral line shifts, the selection of the characteristic peak range in ratio thermometry seriously affects the detection accuracy and sensitivity [40].

To further reduce the range selection error of the two groups of characteristic peaks, we summed the fluorescence images along the short-axis direction to obtain the average fluorescence spectrum. Then we used five different groups of virtual filters, ±0.5 nm, ±1.5 nm, ±4.5 nm, ±7.5 nm, and ±10 nm, to extract the peak intensity and obtain the intensity ratio *FIR* at the corresponding temperature. Finally, each group was fitted to the temperature according to Equation (6) to obtain their respective fitting curves and temperature-related error δ_err_. We plotted their errors together in Figure 8b. The optimal virtual filter is (539 ± 1.5 nm, 806 ± 1.5 nm), of which the maximum temperature error is only 1.1 K. Compared with other groups of virtual filters, the maximum error is reduced by 63% on average. The standard deviation of each group of errors is shown in Table 1.

A narrow filtering range will cause some information on the characteristic peak to be missing, while an excessively wide filtering range will cause crosstalk of the intensity information of the secondary peak, which will affect the temperature measurement accuracy. The high spectral resolution of the reflective spectrometer provides a more accurate virtual filter window for the ratio-based temperature measurement, providing more accurate temperature measurements than commercial 20 nm narrowband filters. After using this virtual filter, the relationship between the *FIR* of the fluorescence spectrum and the temperature *T* is shown in Figure 8c. The judgment coefficient R^2^ of the fitting is 0.9996, which provides an excellent fitting. As shown in Figure 8d, the relative temperature sensitivity *S_r_* of the film layer represents the relative change in the intensity ratio *FIR* when the temperature *T* changes. This indicator has been widely used in various fluorescence thermometers, and its excellent fitting effect has been verified and can be calculated as [14,16,41,42]

(9) $$S_{r}=\left|\frac{1}{FIR}\frac{\partial FIR}{\partial T}\right|\times 100\%$$

Since the carbonization temperature of HPC is 280–300 °C, the theoretical temperature measurement range should be lower than 553.15 K. Figure 8d shows that the temperature sensitivity of the film increases exponentially with the increase in temperature.

In the temperature range of 373.15–508.15 K, the temperature sensitivity reaches up to 2.07%/K, which is mainly due to the opposite temperature dependence curves of the two UCNPs, which increases the rate of change of *FIR*. In addition, the temperature resolution *δ_T_* of the measured temperature can be derived from the following equation [16,43]:

(10) $$\delta_{T}=\frac{1}{S_{r}}\sqrt{{\left(\frac{\delta I_{T,P}}{I_{T,P}}\right)}^{2}+{\left(\frac{\delta I_{T,N}}{I_{T,N}}\right)}^{2}}$$

where *S_r_* is the relative temperature sensitivity of the film layer, and *δI_T_*_,_ *_i/_I_T_*_,_ *_i_* (*i* is *P*, *N*) is the uncertainty of the intensity of the two characteristic peaks, which depends on the performance of the area array sensor in the system. The corresponding uncertainty can be obtained by dividing the fluctuation value of the baseline by the baseline intensity. Once the sensor and characteristic peak values are determined, this value should not fluctuate significantly, and the temperature resolution obtained from the experimental data fitting should be equal to the actual resolution. The average uncertainties of the area array camera for the two sets of peaks in the system are 1.04% and 0.94%, respectively. According to Equations (9) and (10), the temperature resolution is better than 0.028 K, and the best can reach 0.0131 K, which meets the actual temperature measurement needs in the temperature measurement range of 373.15–508.15 K. For specific analysis of temperature resolution and detailed calculation process, please refer to Figure S6 of the Supplementary Materials.

**Figure 9:**
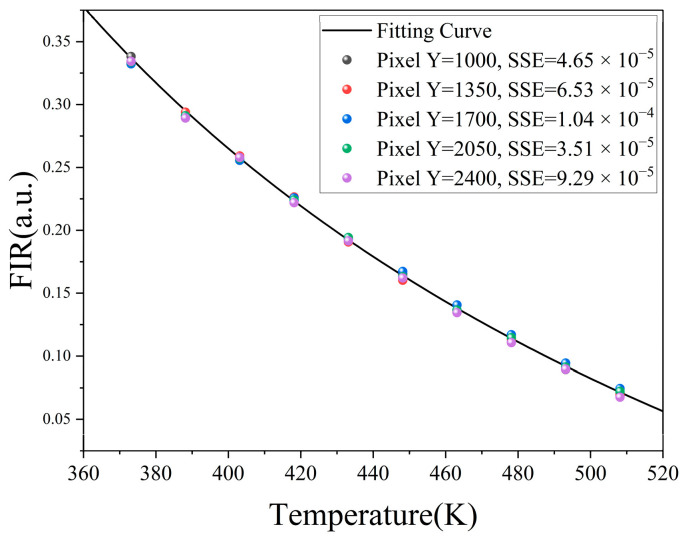
Measured *FIR* values for different pixels and fitting curve.

Finally, it is necessary to examine the influence of the non-linearity of the sensor pixel points on the calibration curve. The non-linear characteristics will cause the intensity of each pixel to be non-linearly proportional to the photons incident on the pixel, thus affecting the detection accuracy [44]. We fit the *FIR*-*T* relationships for the different rows of pixels on CMOS1, respectively, and plotted their fitting curves in Figure 9. The calibration curves of pixels in different rows are basically the same, and the fluctuations in errors in different rows are mainly due to the non-linearity and non-uniformity of pixels. In addition, at high temperatures, the *FIR* value of the edge pixel decreases slightly, mainly because the peak value of the positively quenched material decreases significantly at high temperatures and the uniformity of the line laser also affects the excitation intensity, reducing the SNR, thereby causing the *FIR* to decrease.

### 3.5. Data Fusion and Temperature Inversion

After the calibration work in the previous sections, we obtained the (*y*, *z*) information of the morphology path and the (*y_S_*, *T*) information of the spectral path. Next, we needed to fuse them. Combining Equations (7) and (8) with the distance between the optical axes (1.3988 mm), the relationship between the height coordinate *M_i_* in CMOS2 and the height coordinate *M_Si_* in CMOS1 can be obtained:

(11) $$M_{Si}=\left(\frac{\left(M_{i}-63\right)\omega }{\beta (N_{i})}+1.3988\right)\frac{\beta_{S}(N_{i})}{\omega_{S}}+1738$$

where *β* (*N_i_*) and *β_S_* (*N_i_*) are the object image magnifications of the two modules, and ω and ω_S_ are the pixel sizes of the corresponding sensors.

According to Equation (11), the fusion of (*y*, *z*, *T*) information of each point on the line can be achieved. To realize the reconstruction of the three-dimensional information on the object surface, we also need to control the moving stage to push-broom scan in the direction perpendicular to the *y*-axis and combine the speed v of the moving stage to obtain the width information (*x*-axis). The specific equation is as follows:

(12) $$x_{i}=vt_{i}=\frac{vn_{i}}{\mathrm{FPS}}=\frac{vn_{si}}{{\mathrm{FPS}}_{s}}$$

where *t_i_* is the moving time of the moving stage, *n_i_*, *n_Si_* are the number of photos collected by the two paths, respectively, and FPS, FPS_S_ are the frame rates of CMOS2 and CMOS1, respectively. Due to the different actual frame rates of the two sensors, we can also use Equation (12) to achieve frame synchronization, thereby achieving the effect of sensor synchronization acquisition. After data fusion, the temperature morphology information (*x*, *y*, *z*, *T*) of each point on the surface can be obtained simultaneously.

## 4. Results and Discussion

### 4.1. Verification of Morphology Reconstruction Capability

**Figure 10:**
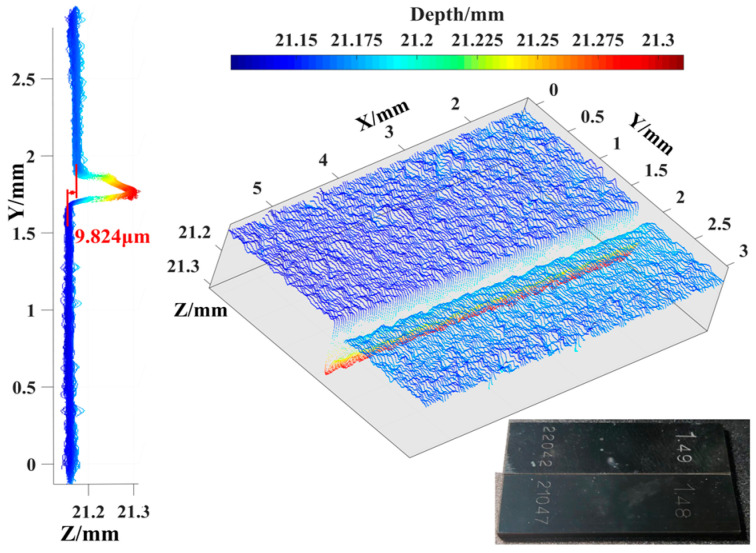
Surface point cloud of 1.48 mm and 1.49 mm standard block gauges; color bars represent distance information.

First, the depth resolution of the MMTL system was verified. As shown in Figure 10, standard block gauges with thicknesses of 1.48 mm and 1.49 mm, respectively, were aligned, and their thickness accuracy was 0.2 μm. Their surfaces were reconstructed using the MMTL system to obtain the corresponding point cloud images. The two surfaces can be clearly distinguished, and the chamfers on both sides of the gauge block can be completely restored. To calculate the change in distance between surfaces, we used the fitting function in Matlab (MathWorks, R2018b, Natick, MA, USA) to fit the two surfaces and we calculated the average distance between the two fitted planes to be 9.824 μm. Compared with the nominal thickness difference of 10 μm, the relative error is 1.76%, which meets the theoretical depth resolution of μm level. The error mainly comes from random read errors of CMOS2 and scratches on the surface; it affects the accuracy of point cloud reconstruction and subsequent fitting in the plane, as shown in Figure 10. The color bar corresponds to the distance from the object point to the equivalent lens plane. The fluctuation range of the single point cloud is about 6.88 μm. Therefore, the minimum depth resolution of the system is 3.44 μm. The error mainly comes from the thickness precision of the standard gauge and surface scratches.

**Figure 11:**
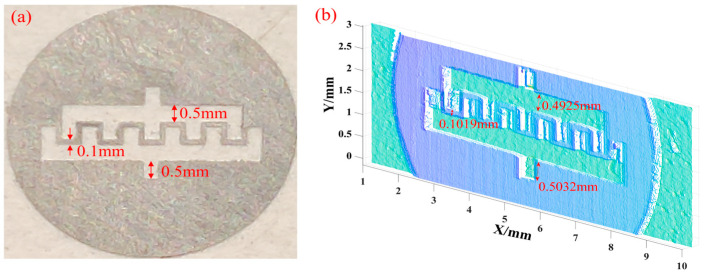
Verification of height resolution. (**a**) FMM. (**b**) Reconstructed point cloud result.

After that, a fine metal mask (FFM) was selected to verify the height resolution of the system, as shown in Figure 11a. This mask (F206, LinChuanJinMi Co., Ltd., Fuzhou, Jiangxi, China) has a diameter of 7 mm, a thickness of 80 μm, and a minimum accuracy of 5 μm, and the narrowest center hollow area is 0.1 mm. It is generally used for interdigital electrode evaporation. As shown in Figure 11b, the system reconstructed the point cloud on the surface of the mask and selected three areas in the height direction to detect the reconstruction effect. The height information of the area can be obtained by calculating the abrupt points at the upper and lower edges of each area and performing a weighted average of the distance between them. The actual heights of the areas are 0.5 mm, 0.5 mm, and 0.1 mm, respectively, the measurement results are 0.4925 mm, 0.5032 mm, and 0.1019 mm, and the relative errors are 1.50%, 0.64%, and 1.9%, respectively. Since the edge of the area is cut by laser, the irregular surface diffusely reflects the laser line and causes errors in the measurement information. The height resolution of the MMTL system at the μm level can significantly reduce the height measurement error.

Finally, the resolution in the *x*-axis depends mainly on the speed of the moving stage. A lower speed increases the system resolution, but the entire scanning period also increases. This moving stage can achieve a moving rate of 0.01 mm/s and a width resolution of up to 0.67 μm (FPS is 15).

**Figure 12:**
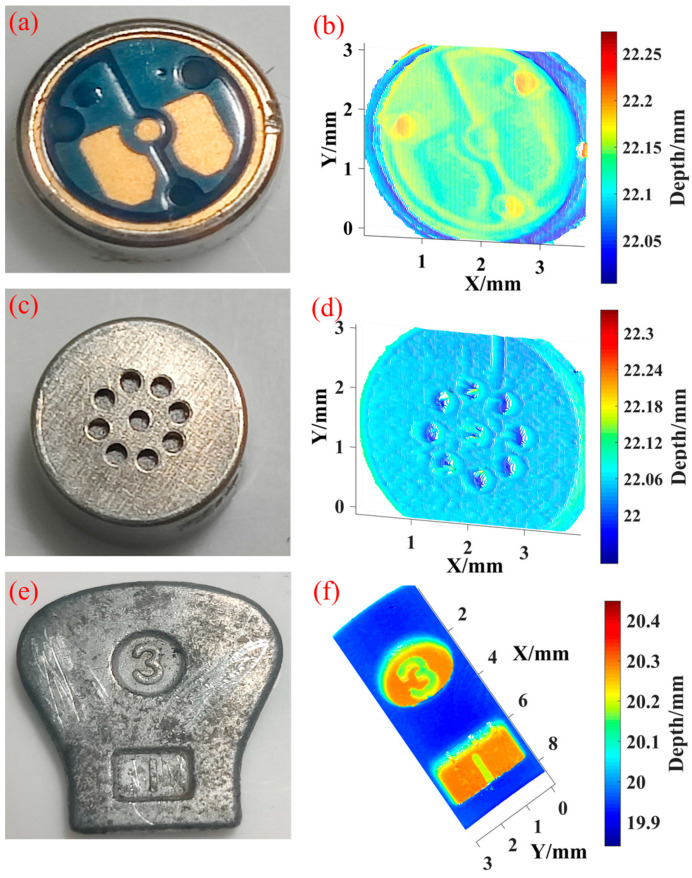
Point cloud reconstruction of three different surface morphologies. (**a**,**c**,**e**) are actual surfaces; (**b**,**d**,**f**) are reconstructed results.

After completing the verification of the system resolution, we selected three objects with different surface morphologies for point cloud reconstruction detection, as shown in Figure 12b,d,f. Figure 12a,c show the two sides of a commercial condenser microphone (EM288Z1, Primo Co., Ltd., Nishitamagun, Japan). The depth of the pressed electrode sheet on the front side is about 50 μm, and the hole diameter on the back side is 0.4 mm. The scratches and chamfers can also be clearly reconstructed. Figure 12e is a zipper puller. The morphological information has large depth changes. The numbers and pits on the surface were accurately reconstructed. The results show that the MMTL system has a good morphological recovery effect, verifying its 3D reconstruction performance.

### 4.2. Temperature Recovery

**Figure 13:**
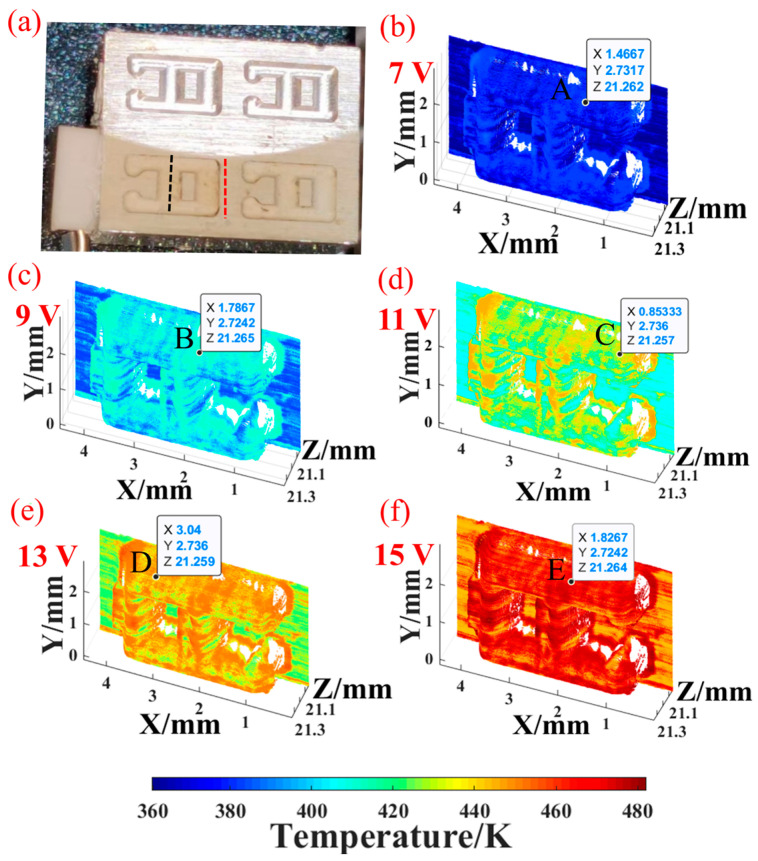
Temperature recovery performance test. Colorbar represents temperature. (**a**) MCH and heated mold; the recovery effect of morphology and temperature measured by *FIR* under different heating voltages: (**b**) 7 V, (**c**) 9 V, (**d**) 11 V, (**e**) 13 V, (**f**) 15 V.

To test the temperature recovery performance of the system, we selected an aluminum alloy mold with a size of 7 mm × 5 mm × 5 mm and attached a metal ceramics heater (MCH) on one side of it using thermal conductive silicone, as shown in Figure 13a. The rated voltage of this MCH is 15 V, and the maximum heating temperature can reach 220 °C. We can adjust the input voltage to control the MCH output power, thereby simulating different operating temperatures of the mold.

The temperature and morphology recovery test of the metal mold was measured at a 2 V interval and the input voltage was 7–15 V. The exposure time of CMOS1 was set to 150 ms and the frame rate was 6.67 fps. The exposure time of the CMOS2 was 50 ms with 20 fps. Therefore, the real frame rate of the system was 6.67 fps. After preprocessing and denoising the collected spectral information, the spectral information of the mold surface was obtained. Using the parameters calibrated in Section 3.4.2 and combined with Equation (6), the temperature information of the mold surface could be restored. After that, the height information y and the width information x were matched using Equations (11) and (12) to obtain the temperature and morphology information of the mold surface, as shown in Figure 13b–f. There was a noticeable temperature gradient between the smooth surface and the groove, so we selected the mold smooth surface area (red line in Figure 13a) and the groove area (black line) to calculate their spatial average temperatures, respectively. The heat flow transmission between the MCH and the metal mold was reflected in the temperature change of the mold surface. After thermal stabilization, the surface temperature changed with the voltage.

In addition, we gave the four-dimensional coordinates (*x*, *y*, *z*, *T*) of several representative groove points and attached the measurement accuracy of the depth of each point, which can verify the four-dimensional information detection performance of the system, as shown in Table 2, where z_real_ is the true depth of the groove.

When the MCH has been working for some time and enters a steady state, the temperature of the mold surface also tends to stabilize. According to the thermal balance theory, the heating power of the MCH is equal to the comprehensive heat dissipation power of the entire heating system. The MCH uses high-temperature materials, such as tungsten, and has pronounced positive temperature coefficient (PTC) characteristics [45]. After heating to the switching temperature, its resistance changes linearly with temperature. Based on Newton’s law of cooling, we can obtain the relationship between the heating voltage *U* and the surface temperature *T* [46]:

(13) $$T\approx \sqrt{\frac{U^{2}}{kK_{t}S}}+b=aU+b$$

where k is the temperature coefficient of MCH, K_t_ is the comprehensive heat dissipation coefficient, S is the heat dissipation area, and a, b are the fitting coefficients.

**Figure 14:**
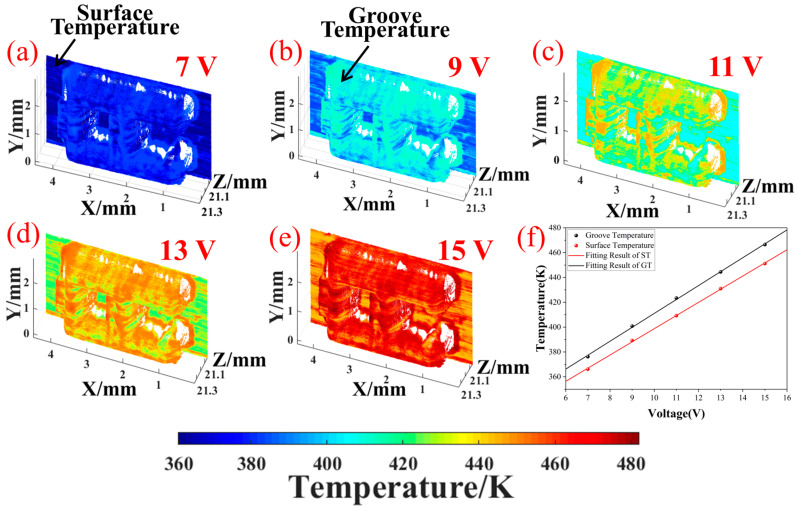
Relationship between the heating voltage and the temperature of two surface regions. (**a**–**e**) are the temperature measurement results of different heating voltages; (**f**) the linear relationship between heating voltage and temperatures in each region.

Figure 14 shows the change in the spatial average temperature of two surface areas under different heating voltages. A strong linear relationship exists between the heating voltage and surface temperature and the fitting determination coefficient R^2^ is 0.9992 and 0.9995, respectively, which is consistent with the theory. Under the same voltage, the temperature at the groove is higher than that at the smooth surface. This may be because the groove area is small (0.7 mm × 1.2 mm) and is not easily exposed to the external environment. The heat radiation may be limited, and the heat in the groove may not be quickly dissipated, resulting in a higher temperature in the groove than on the smooth surface.

**Figure 15:**
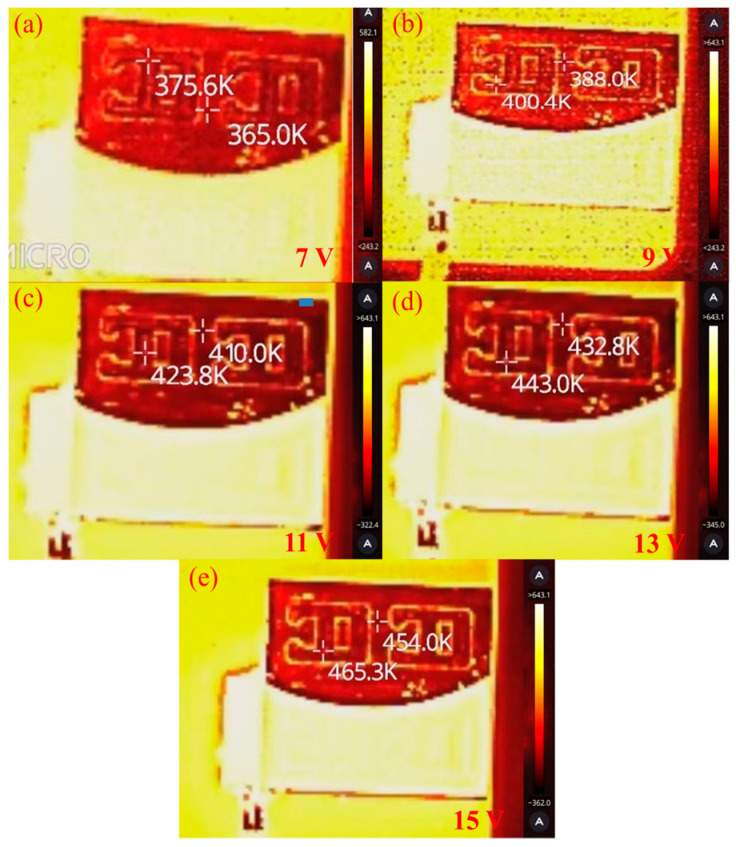
Results of infrared thermometers at different voltages: (**a**) 7 V, (**b**) 9 V, (**c**) 11 V, (**d**) 13 V, (**e**) 15 V.

Figure 15 shows images obtained by a commercial infrared thermometer (P20Max, HIKVISION, Hangzhou, China). Its temperature-measuring range is −20~350 °C, and the measuring accuracy is 1.2 °C. The MMTL system obtained the same measurement results, and the surface temperature gradient was also restored. Due to the large difference between the emissivity of the film layer and the mold material, the infrared thermometer could not simultaneously detect the temperature of the film surface and bare mold, and the application scenario was minimal. In addition, the resolution of this infrared thermometer was only 256 × 192, and high-pixel infrared sensors are often more expensive. Compared with this infrared thermometer, the MMTL system greatly improves the spatial resolution of the system and can capture 3D information of the surface with a more accurate temperature distribution image. Table 3 presents a comparison of the temperature measurement and morphology recovery performance of the MMTL system with existing techniques. For the first time, the MMTL system has improved the spatial resolution of temperature measurement technology and has maintained high temperature sensitivity.

**Figure 16:**
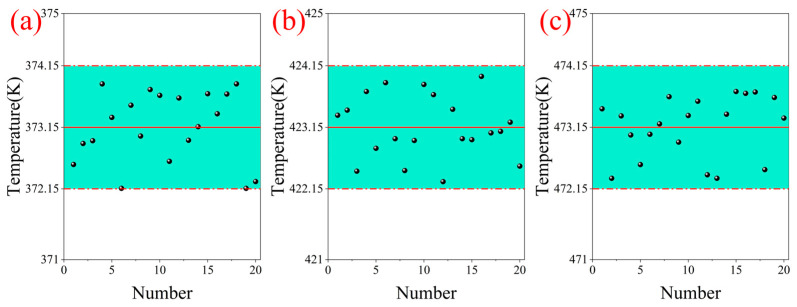
Temperature stability of the system at different detection temperatures: (**a**) 373.15 K, (**b**) 423.15 K, (**c**) 473.15 K.

Finally, we checked the stability of the temperature measurement of the system. Standard block gauges with the exact specifications were selected for thin-film spraying preparation. Three temperature groups of 373.15 K, 423.15 K, and 473.15 K were designed to reduce sampling errors. At each temperature, the temperature information of a random linear area on the film surface was collected multiple times and averaged. The temperature error results of each group are shown in Figure 16. The error of each temperature group is less than 1 K. Their uncertainties at 95% confidence intervals are 373.24 ± 0.2515 K, 423.19 ± 0.2246 K, and 473.21 ± 0.2194 K. In summary, the MMTL system can restore the surface morphology of precision objects while realizing temperature monitoring of each point on the surface. Real-time monitoring of the surface temperature field is also beneficial for accurately reconstructing the temperature gradient and helps to understand the evolution of the surface thermal behavior.

Our system performance can be significantly improved in the following ways. Using a CMOS sensor with high quantum efficiency and low noise can reduce the intensity of the excitation light source [47], thereby increasing the acquisition speed and reducing the thermal effect generated by the laser on the object’s surface. Additional optical groups can be added to the topography optical path for distortion control, further optimizing the detection accuracy in the depth direction [48]. The pixel size of the sensor also determines the spatial resolution. Reducing the pixel size can bring higher spatial resolution. These optimization directions have the potential to significantly improve the performance of our system, paving the way for more accurate and sensitive surface-temperature monitoring and broader applications in lightweight high-resolution sensing systems [49,50] and industrial environments.

**Table 3:** Comparison of technical parameters and performance.

Techniques	Temperature Measurement Performance			3D Morphology Recovery Performance		
	Measurement Range	Temperature Resolution	Error	2D Resolution	Depth Resolution	Depth of Field
MMTL system	373.15 K–508.15 K	0.0131 K	<1 K	2.8 μm	2.1 μm	4.6 mm
Commcial infrared systems (P20Max, HIKVISION, Hangzhou, China)	373.15 K–623.15 K	0.1 K	1.2 K	161.9 μm	-	0.5143 mm
Other microscopic Scheimpflug LiDAR [51]	-	-	-	3.8 μm	3.8 μm	3.5 mm

## 5. Conclusions

In this study, to achieve simultaneous measurement of the three-dimensional morphology and temperature of the surface of micro-objects, we designed and built an MMTL system. The system combines hyperspectral imaging and SLiDAR technology, and has a measurement range of 4.6 mm, a spatial resolution better than 3 μm, a depth resolution of up to 2.1 μm, and a measured error better than 2%, which is widely used in various application scenarios of accurate surface morphology restoration. The spectral imaging module in the system is a self-designed concentric structure reflective spectrometer, which has reasonable aberration control, a theoretical spectral resolution of up to 0.9 nm, and the spatial resolution is better than 3 μm, which is very suitable for the high-precision requirements of *FIR* surface temperature measurement. In addition, we selected a mixed solution of positive and negative quenched upconversion nanomaterials for temperature sensing. After spraying a solid film on the object’s surface, it can achieve a wide temperature range of 373.15 K–508.15 K, with a maximum relative temperature sensitivity of 2.07%/K, and the best temperature resolution is 0.0131 K.

The performance of the MMTL system was verified by restoring the surface morphology of the standard block gauge, FFM, and condenser microphone. The error in the depth direction was about 1.76%, and the error in the height direction was better than 2%. In addition, to detect the system’s temperature recovery performance, the input voltage was used to control the MCH to generate different temperature fields in the micro metal mold, and the MMTL system was used to restore them. The results show that the MMTL system can accurately restore different temperature fields, and the surface temperature distribution also conforms to the heat transfer effect. The heating temperature under different voltages also conforms to Newton’s cooling law. The results of the temperature uncertainty experiment are also less than 1 K. We demonstrated the application scenarios of the system in metal-processing temperature control, integrated circuit thermal management, and mechanical-equipment operation-status monitoring. Its excellent recovery performance has great potential for application in industrial manufacturing and electronic products.

## Figures and Tables

**Table 1:** Standard deviation of each filter group of errors.

Filter Group (nm)	Standard Deviation (K)
±0.5	1.2976
±1.5	0.6651
±4.5	1.2244
±7.5	2.0531
±10	2.6384

**Table 2:** 4D coordinates of several groove points and the measurement accuracy of the depth.

Point #	X/mm	Y/mm	Z/mm	T/K	Z_real_/mm	Z Measurement Accuracy/mm
A	1.467	2.732	21.262	376.19	21.26	0.002
B	1.787	2.724	21.265	400.81		0.005
C	0.853	2.736	21.257	423.39		0.003
D	3.040	2.736	21.259	444.46		0.001
E	1.827	2.724	21.264	466.51		0.004

## Data Availability

The original contributions presented in this study are included in the article/Supplementary Material. Further inquiries can be directed to the corresponding author.

## Supplementary Materials

The following supporting information can be downloaded at: https://www.mdpi.com/article/10.3390/mi16050590/s1, Figure S1: (a) STEM image and (b) EDX spectrum of the NaYF4: 20Yb/2Er@NaYF4 core/shell nanocrystals; Figure S2: (a) SEM image and (b) EDX mappings showing the distribution of elements: (c) O, (d) Tm, (e) Yb, and (f) W in the Tm3+-doped Yb2W3O12 bulk products; Figure S3: Tyndall Effect of nanomaterial colloidal suspension; and Figure S4: Film thickness measurement. (a) Results of two sets of films. (b) Comparison of film thickness uniformity; Figure S5: Thermal effects of 980nm laser, change with laser exposure time; Figure S6: Sum of squares of system intensity uncertainty; Figure S7. Drifts of the collection wavelengths corresponding to the two sets of characteristic spectral lines at different temperatures. SD, Standard Deviation. (a) 373.15 K; (b) 443.15 K; (c) 508.15 K; Table S1: Thickness and flatness of films in different regions.
