# Supplementary material for: High-Resolution Thermometric Scheimpflug LiDAR for Surface Morphology and Temperature Mapping
Source: Micromachines (Basel). 2025 May 18;16(5):590. doi: 10.3390/mi16050590 (PMC12114382; doi:10.3390/mi16050590)
Supplement: Supplementary file 1 [file micromachines-16-00590-s001.zip › micromachines-3602424-supplementary.pdf]

Supplementary Material

1. Luminescent material characterization

$NaYF_4$: 20Yb/2Er@$NaYF_4$ Core/Shell Nanocrystals. The scanning transmission electron microscopy (STEM) image in Figure S1(a) reveals a core-shell structure, with a core of $NaYF_4$: Yb, Er and a shell of $NaYF_4$. The uniformity in the arrangement and size of the particles suggests well-defined core and shell regions. The energy-dispersive X-ray spectroscopy (EDX) spectrum in Figure S1(b) highlights the presence of Sodium (Na), Ytterbium (Yb), Erbium (Er), and Fluorine (F), confirming the core-shell composition, with a significant contribution from Ytterbium and Erbium in the core.

Tm^{3+} Doped $Yb_2W_3O_{12}$ Bulk Products. Figure S2 shows SEM and EDX analysis of $Yb_2W_3O_{12}$ doped with transition metals (Tm). The SEM image (shown in Figure S2(a)) displays the morphology and distribution of the micro clusters, while the EDX spectrum (shown in Figure S2(b)) confirms the elemental composition of $Yb_2W_3O_{12}$, and Tm, validating the successful doping of transition metals and the uniformity of the material.

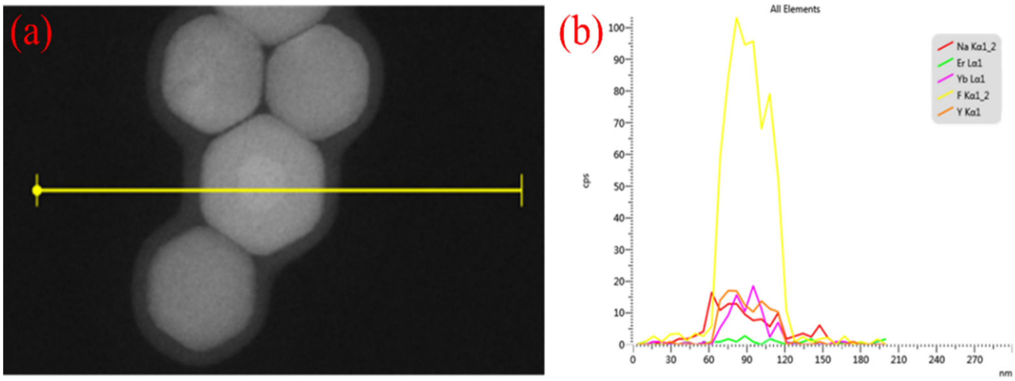

Figure S1. (a) STEM image and (b) EDX spectrum of the $NaYF_4$:20Yb/2Er@$NaYF_4$ core/shell nanocrystals.

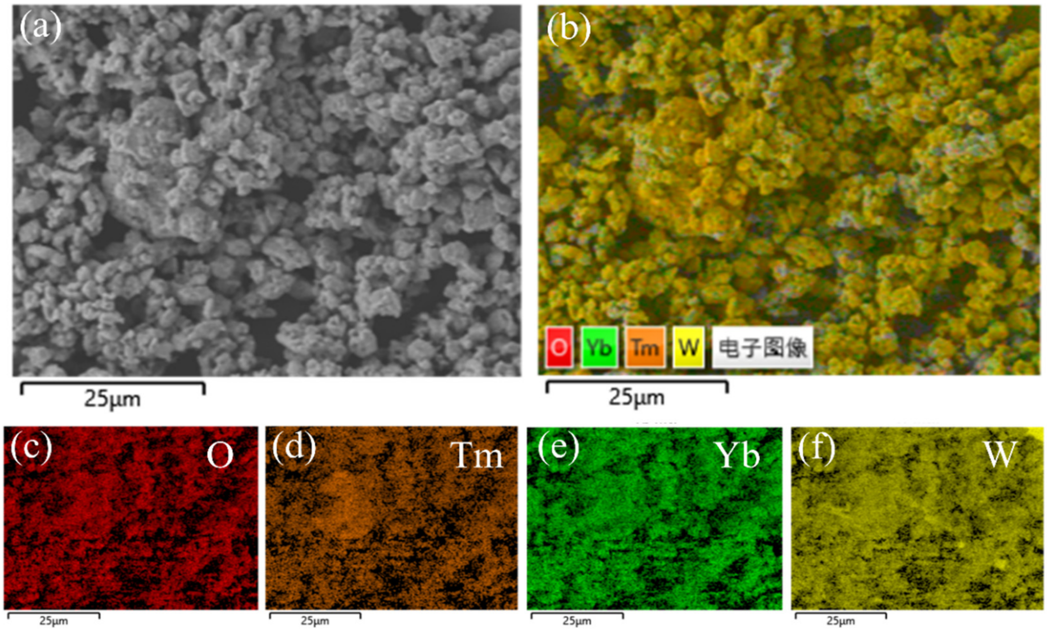

Figure S2. (a) SEM image and (b) EDX mappings showing the distribution of elements: (c) O, (d) Tm, (e) Yb, and (f) W in the Tm^{3+}-doped $Yb_2W_3O_{12}$ bulk products.

2. Colloidal suspension verification and film preparation

As shown in Figure S3, the Tyndall effect can be used to prove that a colloidal suspension has been prepared. The Tyndall effect refers to the fact that when a light beam passes through a colloidal suspension, the light beam becomes visible due to the scattering effect of the colloidal particles, forming a bright light path. By illuminating a light source in a dark environment, the changes in the light as it propagates in the colloidal suspension are observed. If a clear light path appears, it means that the light is scattered by the colloidal particles suspended in the liquid, thereby confirming that the liquid is a colloidal suspension. This phenomenon is different from ordinary solutions (such as ethanol solutions), which do not show obvious light scattering effects.

The prepared colloidal suspension is evenly sprayed onto the surface of the substrate (standard block gauge) using a spray gun (PBQ-350, nVE, Germany). The spraying diameter is 0.2 mm, the spraying air pressure is 300 KPa, and the single spraying time is 3 s. During the spraying process, it should be ensured that the spray covers the surface of the substrate and the distance between the spray gun and the substrate is controlled. The sprayed colloidal suspension forms a liquid film. After spraying the colloidal suspension, the standard block gauge and the liquid film are placed on a heating table for heating. The temperature of the heating table is set to 100 °C to promote the volatilization of the solvent in the colloidal suspension and to form a fixed structure of the colloidal particles on the surface. At this temperature, the film will begin to cure rapidly to form a stable film layer. The heating time needs to be controlled within 5 minutes. Too long a heating time may cause the film layer to be over-cured and uneven. Subsequently, it is cured at room temperature for 1 hour. After the initial curing is completed, in order to improve the adhesion of the film, the film is further heated to 150 °C and maintained for 20 minutes. The purpose of this step is to promote the further cross-linking of HPC molecules and nanomaterials in the film layer through high temperature, thereby enhancing the adhesion of the film to the substrate. Through this series of precisely controlled deposition steps, a relatively uniform and highly adhesive colloidal film can be finally obtained.

3. Film thickness detection

To further analyze the film layer information, we used a step profiler (132192, TMC, America) to measure

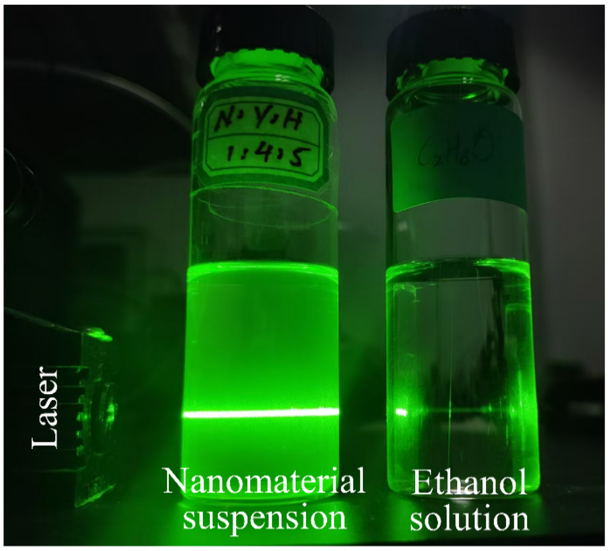

Figure S3. Tyndall Effect of nanomaterial colloidal suspension.

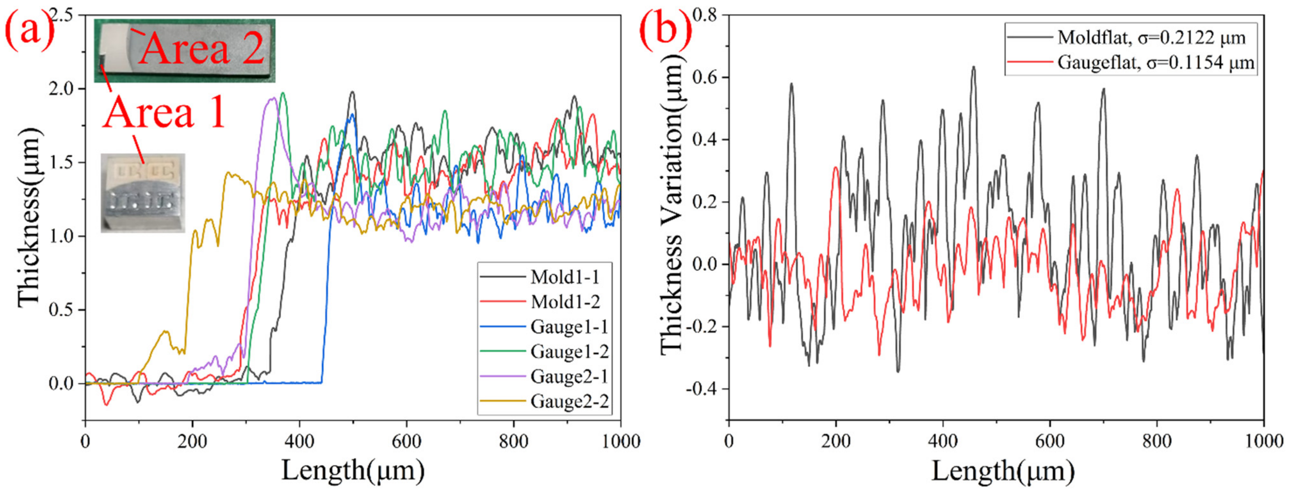

Figure S4. Film thickness measurement. (a) Results of two sets of films. (b) Comparison of film thickness uniformity.

the thickness of the two groups of films in the experiment. The accuracy of this instrument can reach 0.1nm. The two groups of measurement results are shown in Figure S4 (a), and two measurements were taken for each area. The horizontal axis represents the length of the step profiler scanning direction, and the vertical axis represents the film thickness at that location. The film thickness measured in the three regions does not exceed 2 μm, which is less than the system resolution of the MMTL system. For the surface of the object with a pre-coated film, the spatial morphology recovery capability of the MMTL system will not be affected, so that the temperature morphology information of the surface of the object can be restored simultaneously.

Besides, High surface flatness can improve the uniformity of the coated film. On a rough surface, the coating solution may accumulate at high points and form a thinner layer at low points, which will cause uneven thickness of the film. As shown in Figure S4 (b), metal molds with rough surfaces have higher film unevenness. The variation in thickness is referenced to the film thickness at the scanning starting point. Detailed film data are shown in Table S1:

Table S1 Thickness and flatness of films in different regions

Film area	Average film thickness/μm	Film flatness /μm
**Mold 1**	1.627	0.2122
**Gauge 1**	1.562	0.1154
**Gauge 2**	1.238	0.1089

4. Laser Heating Effect

In the temperature recovery experiment, the laser power is 2 W, the single laser exposure time is 10 s, and the push-sweep distance is 4.5 mm. For the polished aluminum alloy mold surface, its specific heat capacity is about 0.95 J/g*K, the weight is 1.8 g, the energy absorption rate under 980 nm irradiation is 6-8%, and the theoretical temperature rise is 0.72 K. To explore the influence of the heating effect of the laser on the mold temperature measurement, we heated the metal mold to 423.15 K and measured the spectral information of the same area on the metal mold after different laser exposure times (2.5 s, 5 s, 10 s). Figure S5 shows the heating effect of the laser, where the horizontal axis is the number of measurement groups and the vertical axis is the temperature result. As the exposure time increases, the temperature measurement results show an upward trend,

and the average temperature increases from 423.33 K to 424.01 K (double-dash line in the figure), but the average temperature difference is less than the temperature measurement error of the MMTL system, which is 0.86 K, and the maximum temperature difference is 0.36% @423.15 K, 1.02% @150 ℃, and the impact on the temperature measurement results can be ignored.

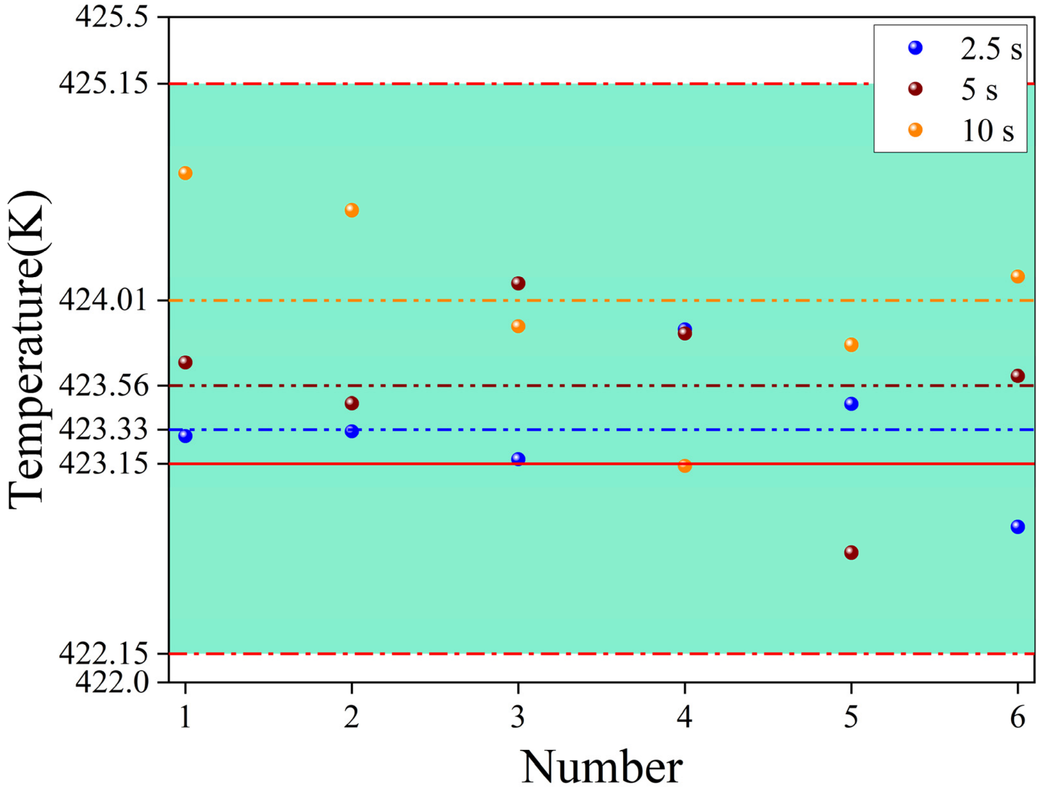

Figure S5. Thermal effects of 976 nm laser, change with laser exposure time.

5. Analysis of system intensity uncertainty

Due to the limitations of the experimental equipment, it is not possible to achieve an experimental environment with an extremely small temperature difference (0.01 K). But we can start with fitting model of experimental data and analyze the factors that affect temperature resolution. As shown in Eq. 10, the temperature resolution δT is mainly affected by the relative temperature sensitivity Sr and the uncertainty of the intensity of the two characteristic peaks ($\delta I_{T,i} / I_{T,i}$).

Since the coefficient of determination (R^2) of the FIR-T relationship fitting model is 0.9996 and the sum of squared error (SSE) is 1.812e-05 (as shown in Figure 8(c)), it indicates a high degree of fitting, and the corresponding relative temperature sensitivity will align with the real model. The uncertainty of the intensity depends on the sensor's detection performance for each set of characteristic peaks, which can be obtained by dividing the fluctuation value of the baseline by the baseline intensity. The resolution of the spectrometer designed in the manuscript can reach 1 nm, which further reduces the impact of reading errors in the

characteristic peaks on the uncertainty of the intensity. When the wavelength range is determined to be ±1.5 nm, the sum of squares of intensity uncertainty (SSU, $\Sigma(\delta I_{T,i} / I_{T,i})^2$) will change with temperature, as shown in Figure S6.

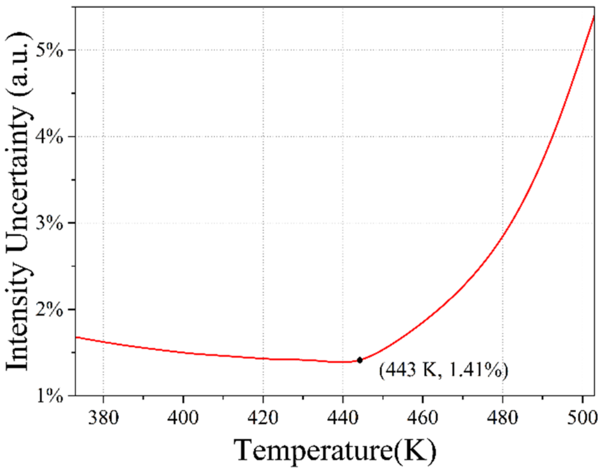

Figure S6. Sum of squares of system intensity uncertainty

When the temperature is 443 K, the minimum SSU of system is 1.41%, and the relative temperature sensitivity is 1.07% /K (as shown in Figure 8(d)). According to Eq. 10, the highest temperature resolution can be calculated as 1.31% K (percent unit), that is 0.0131 K.

Once the sensor and characteristic peak values are determined, this value should not fluctuate significantly. Therefore, the temperature resolution obtained from the experimental data fitting should be equal to the actual resolution.

6. Thermal stability and repetition of system acquisition spectrum

In this experiment, we set the heating stage temperature to three specific values within the system's measurement range (373.15 K, 443.15 K, and 508.15 K) to simulate the impact of varying operational temperatures on spectral data acquisition. A neon calibration light source (NE-1, Wyoptics, China) was selected as the standard spectral reference, with the characteristic spectral lines at 540.06 nm and 811.53 nm chosen to represent the fluorescent peaks near 539 nm and 806 nm, respectively, in the mixed nano-material fluorescence spectrum. These wavelengths were used to investigate the temperature-induced drift of the fluorescence peaks.

The specific experimental steps are as follows: First, we collected two characteristic spectral lines at each temperature every 5 minutes over the course of an hour, followed by heating the system to the next temperature level, and repeating the data collection. Subsequently, the pixel values along the λ-axis corresponding to the characteristic spectral lines in each set of images were extracted, and the difference between these values and those recorded at room temperature was calculated. The wavelength drift was then derived using the wavelength-pixel fitting relationship.

The results in Figure S7 indicate that at an operational temperature of 373.15 K, the wavelength drift for the characteristic spectral lines was less than 0.1 nm, with negligible impact on temperature reconstruction. As the

temperature increased, the wavelength drift slowly increased (for example, the average drift for the 540.06 nm line rose from 0.027 nm to 0.198 nm), but never exceeded 0.35 nm, which is well below the system's spectral resolution of 0.9 nm. Therefore, the drift does not significantly affect the fluorescence intensity readings or the temperature inversion accuracy. Furthermore, the standard deviation of the drift values for the characteristic spectral lines at different temperatures was consistently below 0.09 nm, confirming the stability of the system's spectral information acquisition.

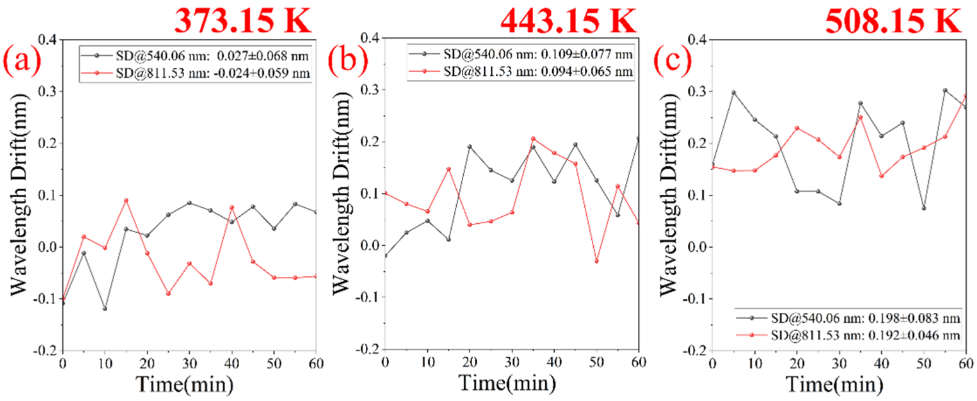

Figure S7. Drifts of the collection wavelengths corresponding to the two sets of characteristic spectral lines at different temperatures. SD, Standard Deviation. (a)373.15 K; (b)443.15 K; (c)508.15 K.
